# Insulin resistance and brain sulcal morphology in non-diabetic adults

**DOI:** 10.1162/IMAG.a.1402

**Published:** 2026-10-16

**Authors:** Daisuke Yoshimaru, Ami Yuzawa, Masakazu Ozawa, Motoki Hirabayashi, Yutaka Matsushita, Tomokazu Tsurugizawa, Hirotaka James Okano

**Affiliations:** Division of Regenerative Medicine, The Jikei University School of Medicine, Tokyo, Japan; National Institute of Advanced Industrial Science and Technology (AIST), Tsukuba, Japan; Department of Radiology, Tokyo Medical University, Tokyo, Japan; Department of Neurology, The Jikei University School of Medicine, Tokyo, Japan; Department of Otorhinolaryngology, The Jikei University School of Medicine, Tokyo, Japan; Institute of Systems and Information Engineering, University of Tsukuba, Tsukuba, Ibaraki, Japan

**Keywords:** insulin resistance, HOMA-IR, sulcal morphology, brain MRI, non-diabetic, frontal lobe

## Abstract

Insulin resistance exerts direct effects on brain structure, yet most neuroimaging evidence comes from populations with established diabetes, and conventional gray matter volumetry may lack sensitivity to detect early structural changes. Here, we examined associations between insulin resistance and sulcal morphology, a measure that integrates cortical thinning, subcortical atrophy, and white matter degeneration into a single outcome, in 728 non-diabetic adults (518 men, 210 women; mean age 61.4 ± 11.6 years). Brain volumes were quantified using OpenMAP-T1, an automated deep learning-based parcellation tool, and associations between HOMA-IR and 30 cerebrospinal fluid-related regional volumes were examined using multiple linear regression adjusted for age, sex, and estimated total intracranial volume, with FDR correction for multiple comparisons. Higher HOMA-IR was significantly associated with greater frontal sulcal volumes (left: β = 596 mm³, right: β = 344 mm³; both FDR-corrected p < 0.001), with a dose–response relationship across insulin resistance categories. This association was observed only in men (sex × HOMA-IR interaction p = 0.019) and was strongest in participants younger than 70 years. Mediation analysis revealed that the relationship between BMI and frontal sulcal enlargement was largely accounted for by the indirect pathway through insulin resistance, with no significant direct effect of BMI. These findings suggest that sulcal morphology can detect metabolic effects on brain structure at a pre-diabetic stage, changes that were not apparent through frontal gray matter volumetry in the same sample, and identify insulin resistance as a potential modifiable target for preserving brain health.

## 1 Introduction

Insulin resistance represents a state of impaired cellular response to insulin signaling that precedes the development of type 2 diabetes mellitus by years to decades. The brain, once considered an insulin-insensitive organ, is now recognized as a major target of insulin action (Kullmann et al., 2016). Insulin receptors are abundantly expressed throughout the central nervous system, with particularly high density in the hippocampus, cerebral cortex, and hypothalamus. Cerebral insulin signaling regulates a broad range of neuronal functions including synaptic plasticity, neurotransmitter release, and neuronal survival. When insulin signaling becomes impaired, these neuroprotective mechanisms are compromised, leading to increased vulnerability to oxidative stress, neuroinflammation, and mitochondrial dysfunction.

The clinical relevance of cerebral insulin resistance is underscored by its association with cognitive impairment and dementia. Prospective cohort studies have demonstrated that elevated insulin resistance, even within the non-diabetic range, predicts accelerated cognitive decline and increased risk of Alzheimer’s disease (Cui et al., 2022). Postmortem studies have revealed reduced insulin receptor expression and impaired downstream signaling cascades in the brains of patients with Alzheimer’s disease, leading some investigators to characterize the condition as “type 3 diabetes” (de la Monte & Wands, 2008). These observations suggest that insulin resistance exerts direct effects on brain structure and function that are independent of, and may precede, the development of frank diabetes.

Magnetic resonance imaging (MRI) studies have provided in vivo evidence linking insulin resistance to brain structural alterations. Higher levels of insulin resistance, typically assessed using the homeostasis model assessment of insulin resistance (HOMA-IR), have been associated with reduced gray matter volumes in the frontal and temporal cortices, hippocampus, and cerebellum (Benedict et al., 2012; Willette et al., 2013). These findings are consistent with the known distribution of insulin receptors in the brain and suggest a pattern of regional vulnerability to metabolic insults. However, gray matter volumetry may lack sensitivity to detect subtle or early stage changes, as substantial neuronal loss must occur before volume reductions become apparent on conventional imaging.

Sulcal morphology offers an alternative approach to assessing brain structural integrity. Cerebral sulci widen as a consequence of cortical thinning, subcortical atrophy, and white matter degeneration, effectively integrating multiple pathological processes into a single measurable outcome. In the aging literature, sulcal enlargement has been shown to correlate with cognitive performance and to predict future cognitive decline, often with greater sensitivity than regional volume measurements (Im et al., 2008; Jin et al., 2018; Liu et al., 2013). The frontal sulci may be particularly informative in the context of metabolic disease, as the prefrontal cortex exhibits high metabolic demand, dense insulin receptor expression, and early involvement in both normal aging and pathological conditions affecting executive function (Kullmann et al., 2015). Sulcal morphology thus represents a biologically grounded and compelling approach for examining how insulin resistance shapes brain structure—one that may capture metabolic effects before they become detectable through conventional gray matter volumetry.

The present study examined the association between insulin resistance and sulcal volumes in non-diabetic adults using automated brain parcellation. We focused on cerebrospinal fluid-related structures including sulci, ventricles, and extra-axial spaces, hypothesizing that higher HOMA-IR would be associated with sulcal enlargement. We further investigated whether any observed associations were modified by age and sex, and whether body mass index mediated the relationship between insulin resistance and brain structure.

## 2 Methods

### 2.1 Study participants

This retrospective cross-sectional study was approved by the Institutional Review Board of The Jikei University School of Medicine (approval number: 36-237(12346)) and was conducted in accordance with the 1964 Declaration of Helsinki and its later amendments. The requirement for written informed consent was waived due to the retrospective nature of the study. Participants were recruited from individuals who underwent voluntary health examinations at the health checkup center affiliated with The Jikei University School of Medicine. This center provides comprehensive medical checkups (ningen dock) to the general public on a voluntary, fee-based basis and is not restricted to occupational or hospital-based populations. Data were collected between April 2022 and July 2024. Participants were provided the opportunity to opt out through a public notice displayed at the institution. From an initial cohort of 957 individuals, we excluded participants with missing MRI data (n = 1), a history of diabetes mellitus (n = 38), cerebrovascular disease (n = 8), other brain-related disorders including brain tumors, traumatic brain injury, and neurodegenerative diseases (n = 10), and missing HOMA-IR data (n = 172). The final analytical sample consisted of 728 non-diabetic adults (518 men and 210 women) aged 22 to 92 years (Fig. 1).

**Fig. 1.**
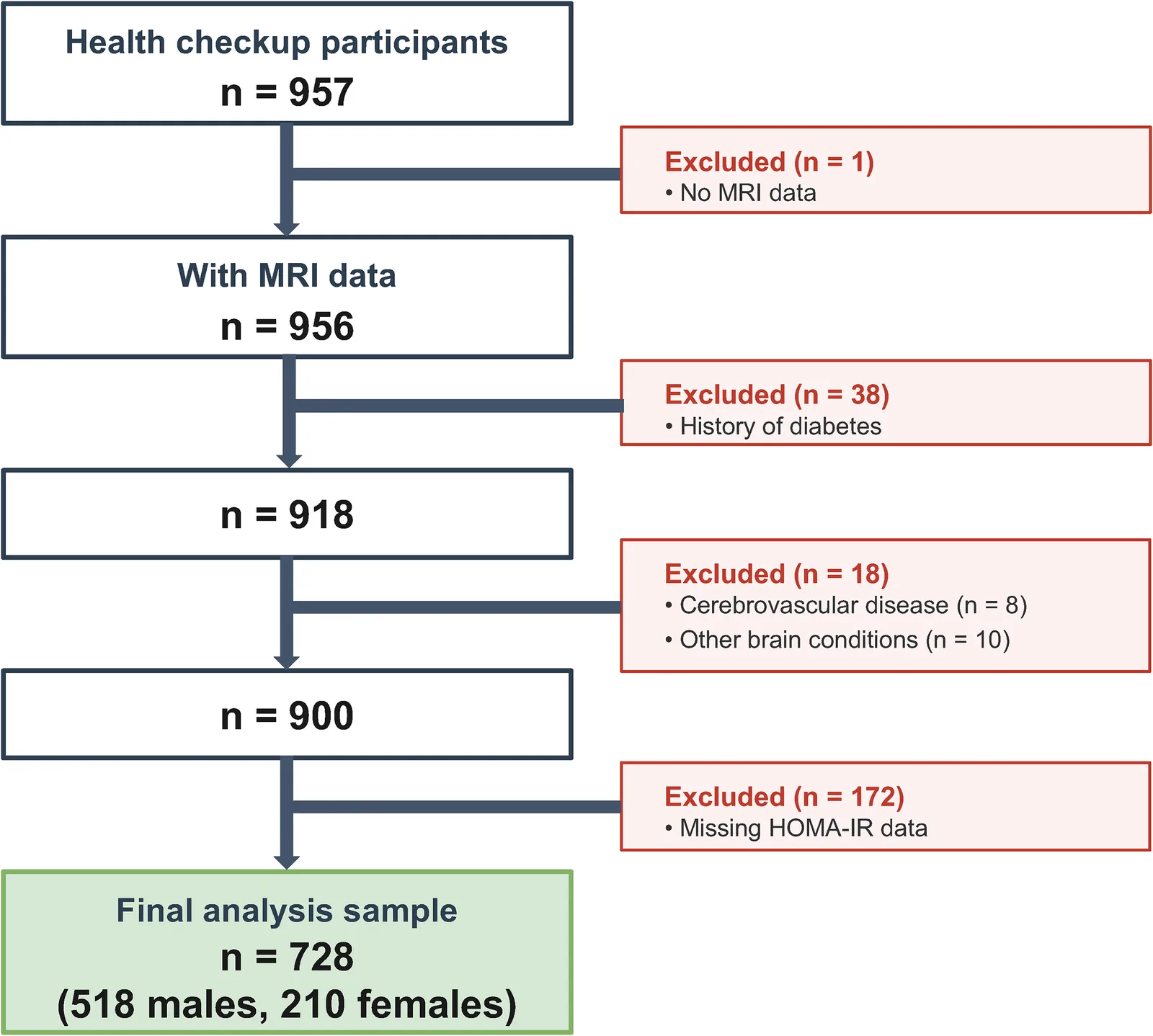
Participant flow diagram. Of 957 health checkup participants, 728 were included in the final analysis after excluding those with no MRI data (n = 1), a history of diabetes mellitus (n = 38), cerebrovascular disease or other brain conditions (n = 18), and missing HOMA-IR data (n = 172). The final sample comprised 518 men and 210 women.

### 2.2 Brain MRI acquisition and processing

Brain MRI scans were acquired using a 3 Tesla MRI scanner (Siemens Skyra) with a standardized T1-weighted three-dimensional magnetization-prepared rapid gradient echo (MPRAGE) sequence. Imaging parameters included repetition time = 1800 ms, echo time = 2.85 ms, inversion time = 800 ms, flip angle = 10°, and isotropic voxel size = 1 × 1 × 1 mm.

Automated brain segmentation was performed using OpenMAP-T1, a deep learning-based parcellation tool that segments the brain into 144 anatomical regions (Nishimaki et al., 2024). The regions of interest for the primary analysis included 30 cerebrospinal fluid-related structures comprising 18 sulcal regions (frontal, central, Sylvian, parietal, cingulate, occipital, and temporal sulci, bilaterally), 8 ventricular regions (anterior, posterior, and inferior lateral ventricles bilaterally, third ventricle, and fourth ventricle), and 4 extra-axial cerebrospinal fluid spaces (anterior and posterior, bilaterally). Volumetric data were extracted in cubic millimeters. Estimated total intracranial volume was calculated as the sum of all 144 regional volumes. Preprocessing, including N4 bias-field correction, was performed automatically within the OpenMAP-T1 pipeline prior to parcellation. OpenMAP-T1 generates an automated quality control flag for each case by comparing the volumes of eight major brain divisions against the distribution observed in the tool’s training datasets, flagging values that fall outside the training range. No cases in the present sample were flagged as falling outside this range. In addition to this automated quality control, all parcellation outputs were independently inspected by two of the authors (D.Y. and A.Y.) to confirm anatomical plausibility. Rather than applying predefined quantitative thresholds, this visual review focused on identifying obvious segmentation failures—such as gross tissue misclassification or anatomically implausible regional boundaries—which served as the criterion for exclusion. This visual assessment was performed on the basis of the parcellation images alone, without reference to participants’ clinical or metabolic information, and was intended to complement, rather than replace, the tool’s built-in automated quality control. No cases required exclusion on the basis of this review. A representative example of whole-brain parcellation is shown in Supplementary Figure S3. Validation of OpenMAP-T1 against expert manual labels has demonstrated strong volumetric agreement across regions (Pearson and Spearman correlations >0.95; Nishimaki et al., 2024). Dice similarity coefficients for sulcal and CSF-related regions (0.62–0.75) are somewhat lower than for cortical and white matter regions (0.69–0.79), reflecting greater inter-individual variability in sulcal boundary definition rather than systematic measurement error; as the present analyses examined relative associations rather than absolute volumes, this is unlikely to have materially affected our findings.

### 2.3 Assessment of insulin resistance

Fasting blood samples were collected during health examinations. Fasting plasma glucose and fasting serum insulin were measured, and the HOMA-IR was calculated as fasting insulin (μU/mL) × fasting glucose (mg/dL)/405 (Matthews et al., 1985). For categorical analyses, participants were classified into three groups based on established cutoff values (normal, HOMA-IR <1.6; borderline, 1.6–2.5; insulin resistant, ≥2.5) and into quartiles based on the sample distribution (Yamada et al., 2011, 2012). These cutoffs were derived from non-diabetic Japanese adults, making them directly applicable to the present cohort in terms of both ethnicity and diabetes status.

### 2.4 Covariates

Demographic and clinical data were obtained from health examination records. Age was recorded in years, and sex was coded as a binary variable. Body mass index (BMI) was calculated as weight in kilograms divided by height in meters squared. Systolic blood pressure, serum triglycerides, and high-density lipoprotein cholesterol were obtained from health examination records.

### 2.5 Statistical analysis

All statistical analyses were performed using Python 3.10 with the statsmodels, scipy, and pandas libraries. Continuous variables were summarized as means with standard deviations, and categorical variables were summarized as counts with percentages.

The primary analysis examined associations between HOMA-IR and regional brain volumes using multiple linear regression models. Each regional volume was modeled as the dependent variable, with HOMA-IR as the primary independent variable and age, sex, and estimated total intracranial volume as covariates. Unstandardized regression coefficients (β) with 95% confidence intervals were reported, representing the change in regional volume (in mm³) per unit increase in HOMA-IR. Standardized regression coefficients were also calculated for comparison across regions. To account for multiple comparisons across 30 cerebrospinal fluid-related regions, p-values were adjusted using the Benjamini–Hochberg false discovery rate method, with statistical significance set at FDR-corrected p < 0.05.

Categorical analyses compared regional volumes across HOMA-IR groups using one-way analysis of variance with post-hoc Tukey tests. Dose–response relationships were evaluated using Spearman rank correlation between HOMA-IR quartile rank and regional volumes. The percentage difference between the highest and lowest quartiles was calculated to provide clinical context.

Sex-stratified analyses were performed by fitting separate regression models for men and women. To formally test for sex differences, interaction models were constructed including a HOMA-IR × sex interaction term, with the significance of the interaction coefficient indicating effect modification by sex.

Age-stratified analyses were performed by dividing participants into three groups (younger than 55 years, 55 to 69 years, and 70 years or older) and fitting regression models within each stratum. Given the narrower age range within strata, age was not included as a covariate in these stratified models.

Mediation analysis was conducted to examine whether HOMA-IR mediates the association between BMI and frontal sulcal volume. The analysis followed the Baron and Kenny framework (Baron & Kenny, 1986), estimating the total effect of BMI on brain volume (path c), the effect of BMI on HOMA-IR (path a), the effect of HOMA-IR on brain volume adjusted for BMI (path b), and the direct effect of BMI adjusted for HOMA-IR (path c′). The indirect effect (a × b) was tested using bootstrapping with 5000 resamples, and the proportion mediated was calculated as the indirect effect divided by the total effect.

Sensitivity analyses were performed to assess the robustness of the findings. These included excluding participants with extreme HOMA-IR values (greater than 5), excluding participants older than 80 years, additional adjustment for systolic blood pressure, triglycerides, and high-density lipoprotein cholesterol, restricting the analysis to participants with normal BMI (less than 25 kg/m²), and restricting to participants younger than 70 years.

Supplementary analyses examined associations between HOMA-IR and cortical and subcortical gray matter volumes in 26 regions previously implicated in insulin resistance, including frontal, temporal, parietal, and occipital cortices, hippocampus, amygdala, thalamus, insula, cingulate cortex, basal ganglia, and cerebellum.

## 3 Results

### 3.1 Participant characteristics

The study included 728 non-diabetic adults (518 men and 210 women) with a mean age of 61.4 ± 11.6 years (range 22–92). Mean BMI was 23.4 ± 3.5 kg/m², and mean HOMA-IR was 1.33 ± 0.90 (range 0.31–8.90). Based on established cutoff values, 532 participants (73.1%) were classified as normal (HOMA-IR <1.6), 127 (17.4%) as borderline (1.6–2.5), and 69 (9.5%) as insulin resistant (≥2.5). Men had significantly higher HOMA-IR than women (1.45 ± 0.96 vs 1.05 ± 0.69, p < 0.001). The mean estimated total intracranial volume was 1,332,860 ± 123,733 mm³, with men having larger volumes than women (1,379,304 vs 1,218,297 mm³, p < 0.001) (Table 1).

**Table 1.** Participant characteristics.

Variable	Men (n = 518)	Women (n = 210)
Age, years	61.4 ± 11.4	61.4 ± 12.2
BMI, kg/m²	24.2 ± 3.3	21.5 ± 3.4
HOMA-IR	1.45 ± 0.96	1.05 ± 0.69
Median (IQR)	1.19 (0.81–1.84)	0.82 (0.60–1.25)
HOMA-IR categories, n (%)
Normal (<1.6)	353 (68.1)	179 (85.2)
Borderline (1.6–2.5)	107 (20.7)	20 (9.5)
Insulin resistant (≥2.5)	58 (11.2)	11 (5.2)

Data are presented as mean ± SD, median (IQR), or n (%).

BMI, body mass index; HOMA-IR, homeostatic model assessment for insulin resistance; IQR, interquartile range.

### 3.2 Association between HOMA-IR and cerebrospinal fluid-related brain volumes

Multiple linear regression analyses adjusted for age, sex, and estimated total intracranial volume revealed significant positive associations between HOMA-IR and 6 of the 30 cerebrospinal fluid-related regions after FDR correction. The strongest associations were observed in the frontal sulci. Each unit increase in HOMA-IR was associated with an increase of 596.2 mm³ in left frontal sulcal volume (95% CI 372 to 821, FDR-corrected p < 0.001) and 343.5 mm³ in right frontal sulcal volume (95% CI 181 to 506, FDR-corrected p < 0.001). Significant positive associations were also found in the right anterior extra-axial cerebrospinal fluid space (β = 307.4 mm³, 95% CI 139 to 476, FDR-corrected p = 0.004), left anterior extra-axial space (β = 269.8 mm³, 95% CI 98 to 441, FDR-corrected p = 0.013), and right posterior extra-axial space (β = 246.9 mm³, 95% CI 90 to 404, FDR-corrected p = 0.013). The left cingulate sulcus also showed a significant association (β = 78.3 mm³, 95% CI 20 to 136, FDR-corrected p = 0.042). No significant associations were observed between HOMA-IR and any of the eight ventricular regions (Supplementary Fig. S1).

### 3.3 Dose–response relationship

Categorical analyses demonstrated a dose–response relationship between HOMA-IR and frontal sulcal volumes. Mean left frontal sulcal volume increased progressively across HOMA-IR categories (normal 15,335 mm³, borderline 15,839 mm³, insulin resistant 16,586 mm³; p for trend < 0.001). A similar pattern was observed for right frontal sulcal volume (normal 11,268 mm³, borderline 11,657 mm³, insulin resistant 12,060 mm³; p for trend < 0.001) (Fig. 2). Quartile analyses showed that participants in the highest HOMA-IR quartile (1.69–8.90) had 8.9% greater left frontal sulcal volume and 7.9% greater right frontal sulcal volume than those in the lowest quartile (0.31–0.73), corresponding to absolute differences of 1,767 mm³ and 1,152 mm³, respectively (both p < 0.001).

**Fig. 2.**
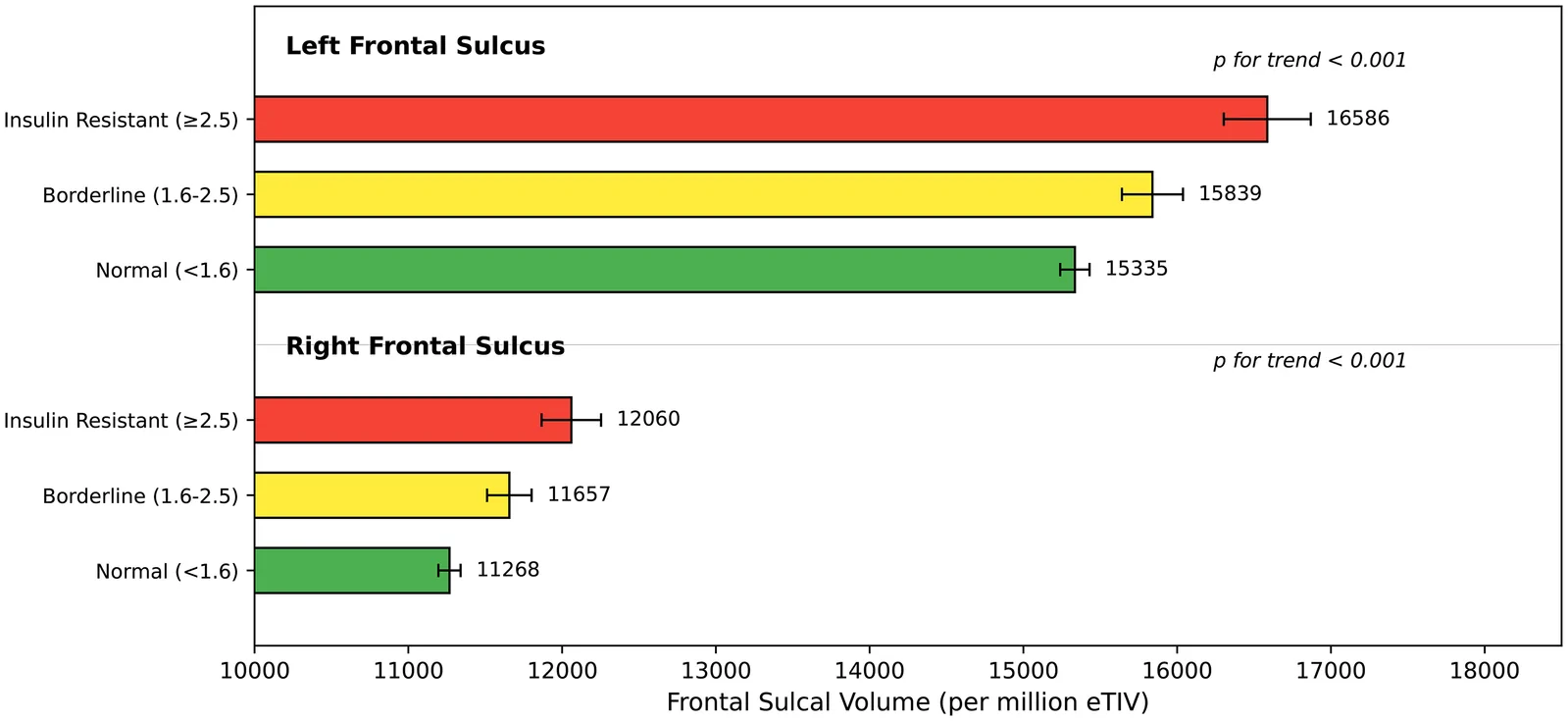
Association between HOMA-IR categories and frontal sulcal volume. Mean frontal sulcal volumes (adjusted for estimated total intracranial volume) are shown for each HOMA-IR category: normal (<1.6), borderline (1.6–2.5), and insulin resistant (≥2.5). Error bars represent standard error of the mean. Both left and right frontal sulcal volumes increased progressively with higher HOMA-IR categories (p for trend < 0.001 for both).

### 3.4 Sex differences

Sex-stratified analyses revealed that the associations between HOMA-IR and brain volumes were present only in men. In men, HOMA-IR was significantly associated with left frontal sulcal volume (β = 725.1 mm³, 95% CI 475 to 975, p < 0.001) and right frontal sulcal volume (β = 416.0 mm³, 95% CI 238 to 594, p < 0.001). In women, no significant associations were observed (left frontal sulcus β = 156.1 mm³, p = 0.55; right frontal sulcus β = 126.7 mm³, p = 0.52) (Fig. 3). Formal interaction testing confirmed significant effect modification by sex for the left frontal sulcus (interaction p = 0.019), right anterior extra-axial space (interaction p = 0.030), right posterior extra-axial space (interaction p = 0.010), and left anterior extra-axial space (interaction p = 0.021).

**Fig. 3.**
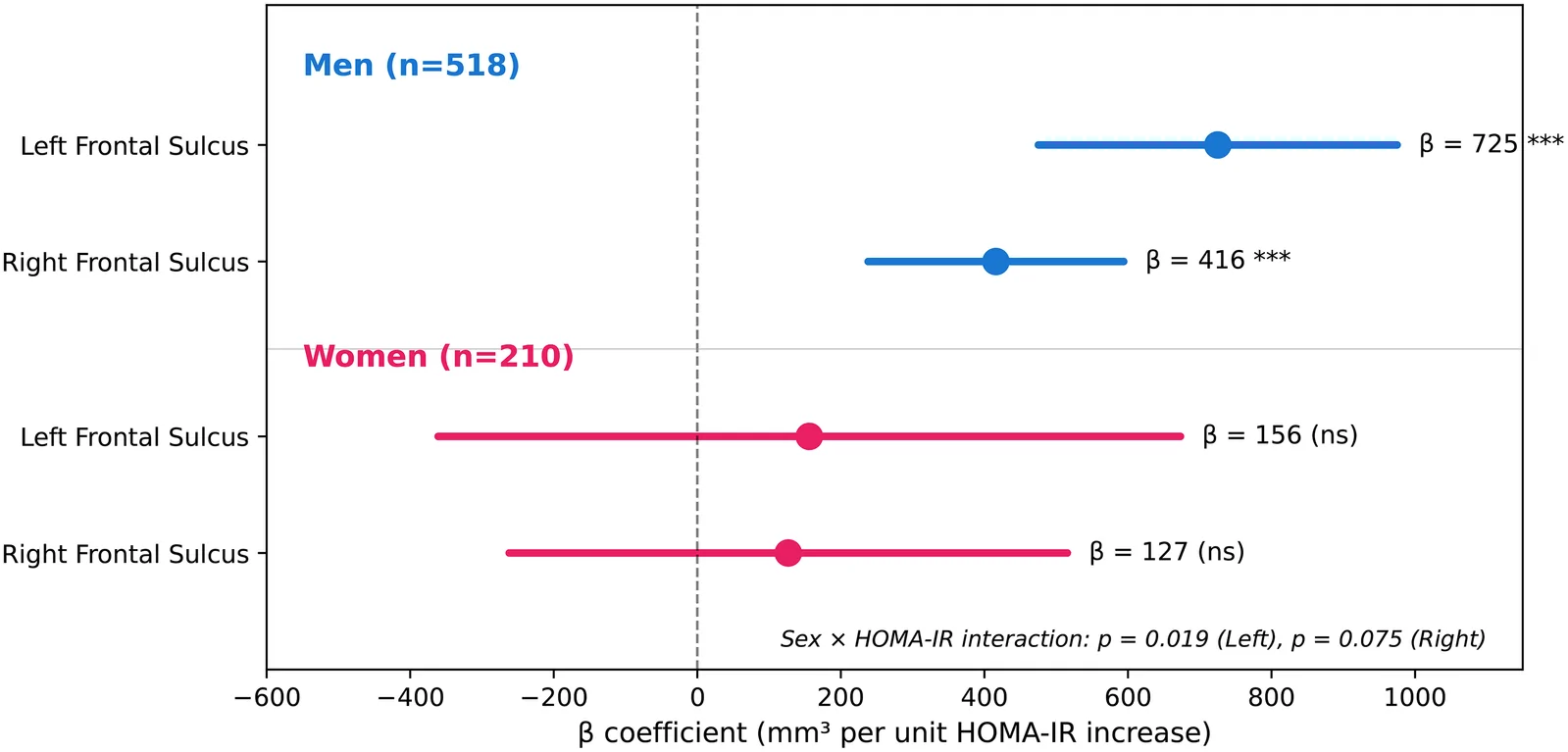
Sex differences in the association between HOMA-IR and frontal sulcal volume. Forest plot showing β coefficients (with 95% confidence intervals) for the association between HOMA-IR and frontal sulcal volume, stratified by sex. Associations were significant in men (n = 518) but not in women (n = 210). Sex × HOMA-IR interaction was significant for the left frontal sulcus (p = 0.019) and marginally significant for the right frontal sulcus (p = 0.075). Models were adjusted for age and estimated total intracranial volume. ***p < 0.001; ns, not significant.

### 3.5 Age-stratified analyses

The association between HOMA-IR and frontal sulcal volume varied by age group. In participants younger than 55 years (n = 188), each unit increase in HOMA-IR was associated with an increase of 784.6 mm³ in left frontal sulcal volume (95% CI 370 to 1199, p < 0.001). In participants aged 55 to 69 years (n = 369), the association remained significant (β = 618.3 mm³, 95% CI 298 to 938, p < 0.001). However, in participants aged 70 years or older (n = 171), the association did not reach statistical significance (β = 529.1 mm³, 95% CI −4 to 1062, p = 0.052). Similar patterns were observed for the right frontal sulcus (Supplementary Fig. S2).

### 3.6 Mediation analysis

Mediation analysis examined whether HOMA-IR mediates the relationship between BMI and frontal sulcal volume. The total effect of BMI on left frontal sulcal volume was significant (c = 86.4 mm³ per kg/m², p = 0.006). BMI was strongly associated with HOMA-IR (path a, β = 0.162, p < 0.001), and HOMA-IR was associated with left frontal sulcal volume after adjusting for BMI (path b, β = 634 mm³, p < 0.001). The direct effect of BMI on left frontal sulcal volume became non-significant after adjusting for HOMA-IR (c′ = −16.6 mm³, p = 0.67). The indirect effect through HOMA-IR was significant (a × b = 103.0 mm³, 95% CI 60 to 149, p < 0.001), with a proportion mediated of 119% (Fig. 4). These findings are consistent with insulin resistance largely accounting for the statistical association between BMI and frontal sulcal volume.

**Fig. 4.**
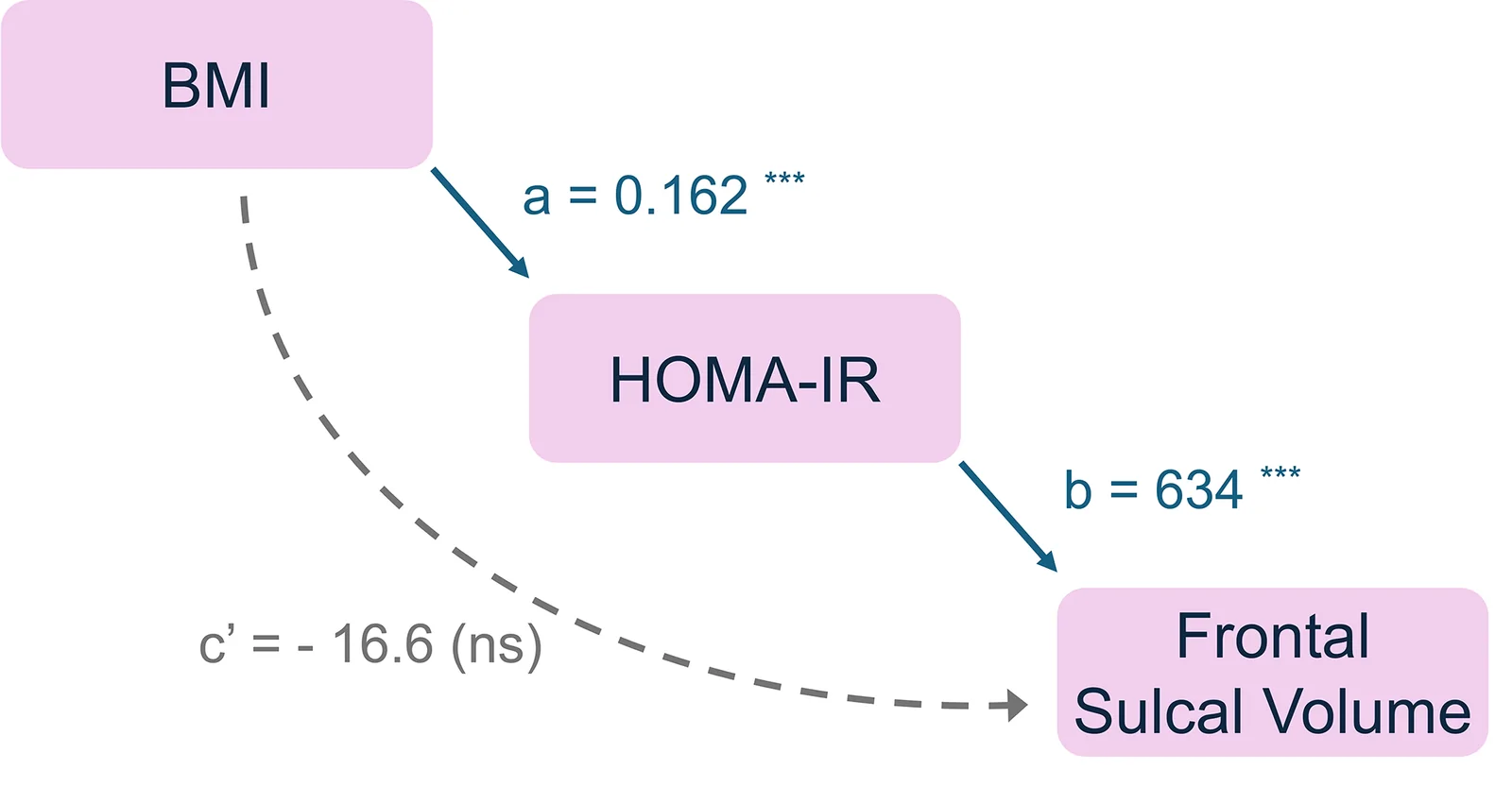
Mediation analysis of the relationship between BMI, HOMA-IR, and frontal sulcal volume. Path diagram illustrating the mediation of the BMI–frontal sulcal volume relationship by HOMA-IR. Path a represents the effect of BMI on HOMA-IR; path b represents the effect of HOMA-IR on frontal sulcal volume (adjusted for BMI); path c′ represents the direct effect of BMI on frontal sulcal volume (adjusted for HOMA-IR). The indirect effect (a × b = 103 mm³, 95% CI: 60–149, p < 0.001) was significant, while the direct effect (c′ = −16.6 mm³, p = 0.67) was not statistically significant. The proportion mediated was 119%, exceeding 100% due to a suppression effect in which the direct and indirect effects were opposite in sign. All models were adjusted for age, sex, and estimated total intracranial volume. ***p < 0.001; ns, not significant.

### 3.7 Sensitivity analyses

The association between HOMA-IR and frontal sulcal volume remained significant after excluding 3 participants with extreme HOMA-IR values greater than 5 (left frontal sulcus β = 656.0 mm³, p < 0.001), after excluding 38 participants older than 80 years (β = 599.7 mm³, p < 0.001), and after additional adjustment for systolic blood pressure, triglycerides, and high-density lipoprotein cholesterol (β = 551.5 mm³, p < 0.001). The association was stronger when restricted to participants younger than 70 years (β = 655.5 mm³, p < 0.001). However, when the analysis was restricted to 511 participants with normal BMI (less than 25 kg/m²), the association was no longer significant (β = 385.0 mm³, 95% CI −30 to 800, p = 0.07). HOMA-IR was both lower and less variable among normal-BMI participants (mean ± SD, 1.05 ± 0.57) than among overweight/obese participants (2.00 ± 1.15), and only 14 of 511 normal-BMI participants (2.7%) met criteria for insulin resistance (HOMA-IR ≥2.5), compared with 55 of 217 (25.3%) overweight/obese participants (Supplementary Table S1). The association remained significant after additional adjustment for lifestyle-related factors, including smoking status, alcohol consumption, physical activity, regular exercise, and sleep recovery (β = 641.0 mm³, 95% CI 334 to 948, p < 0.001; n = 471). This sensitivity analysis excluded 257 participants with missing data on alcohol consumption (n = 252), physical activity (n = 9), regular exercise (n = 3), or sleep recovery (n = 12); some participants had missing data on more than one variable.

### 3.8 Cortical and subcortical gray matter volumes

Supplementary analyses of 26 cortical and subcortical regions revealed significant negative associations between HOMA-IR and gray matter volumes in 4 regions after FDR correction. Each unit increase in HOMA-IR was associated with a decrease of 573.5 mm³ in right cerebellar gray matter (95% CI −900 to −247, FDR-corrected p = 0.011) and 540.3 mm³ in left cerebellar gray matter (95% CI −870 to −211, FDR-corrected p = 0.017). Significant negative associations were also observed in the right fusiform gyrus (β = −140.9 mm³, 95% CI −219 to −63, FDR-corrected p = 0.011) and left fusiform gyrus (β = −112.7 mm³, 95% CI −189 to −36, FDR-corrected p = 0.036). No significant associations were found for the hippocampus, thalamus, frontal cortex, or other regions examined.

## 4 Discussion

This study provides the first evidence that insulin resistance is associated with subtle structural brain changes at a pre-diabetic stage. Critically, these changes became detectable through sulcal morphology—an approach that may offer greater sensitivity than conventional gray matter volumetry for capturing early metabolic effects on the brain. The association exhibited a clear dose–response relationship, and mediation analysis suggested that insulin resistance, rather than adiposity itself, largely accounted for this relationship. Additionally, we identified cerebellar gray matter as another region vulnerable to insulin resistance. These findings suggest that insulin resistance represents a modifiable target for preventing effects on brain structure, with potential implications for the timing of interventions aimed at preserving brain health.

### 4.1 Interpretation of frontal sulcal findings

A notable finding of this study is that insulin resistance was associated with frontal sulcal enlargement but not with frontal gray matter volume. This apparent discrepancy may be explained from both methodological and biological perspectives.

From a methodological standpoint, previous studies have suggested that sulcal measures may be more sensitive to early brain changes than conventional volumetric measures. Im and colleagues reported that among four morphological measures, sulcal widening showed the highest sensitivity in differentiating mild cognitive impairment from normal controls (Im et al., 2008). Liu and colleagues found in a longitudinal study that sulcal changes were accelerated in individuals with mild cognitive impairment, whereas cortical thickness changes were similar across cognitive groups (Liu et al., 2013). Several factors may contribute to the higher sensitivity of sulcal measures. First, sulcal measurements rely on the gray matter–cerebrospinal fluid contrast rather than the gray–white matter contrast, and are not dependent on the accurate identification of gray–white matter borders, which may improve measurement reliability (Jin et al., 2018). Second, from a statistical perspective, sulcal volumes have smaller absolute values at baseline than gray matter volumes. Consequently, the same absolute change represents a larger proportional change for sulci than for gray matter, resulting in greater statistical power to detect group differences. This differential sensitivity could partly explain why sulcal measures detected insulin resistance-related changes in our study.

From a biological standpoint, recent evidence has linked insulin resistance to enlarged perivascular spaces in non-diabetic elderly populations (Wu et al., 2020). Enlarged perivascular spaces are considered an imaging marker of glymphatic system dysfunction, and insulin resistance has been reported to correlate negatively with glymphatic function (Tuerxun et al., 2024). Since perivascular spaces are cerebrospinal fluid-filled compartments, their enlargement could potentially contribute to increased sulcal volume. Whether the frontal sulcal enlargement observed in our study reflects glymphatic dysfunction remains speculative and warrants investigation in future studies incorporating direct measures of glymphatic function. A significant association was also observed between HOMA-IR and left cingulate sulcal volume (β = 78.3 mm³, FDR-corrected p = 0.042), though this was the weakest of the six significant findings and was not replicated in the right hemisphere. This finding is broadly consistent with prior reports linking insulin resistance to structural changes in the cingulate cortex: Cao and colleagues reported that insulin resistance severity was inversely associated with cortical thickness in the rostral anterior cingulate gyrus in individuals with type 2 diabetes (Cao et al., 2024), and a systematic review identified the left anterior cingulate as one of the most consistently affected regions across cortical thickness studies in type 2 diabetes (Motaghi et al., 2024). As cortical thinning and sulcal enlargement reflect complementary manifestations of the same underlying structural change, the present finding extends this evidence to a non-diabetic population. The marginal FDR-corrected p-value and absence of a bilateral pattern nonetheless warrant cautious interpretation.

### 4.2 Cerebellar and other gray matter findings

The significant association between insulin resistance and reduced cerebellar gray matter volume is consistent with emerging evidence of cerebellar vulnerability to metabolic disturbances. The cerebellum has a particularly high density of insulin receptors and extracts glucose from the circulation efficiently, potentially making it susceptible to insulin signaling dysfunction (Kleinridders et al., 2014). Previous studies have reported cerebellar structural abnormalities in patients with type 2 diabetes (Chen et al., 2024; Oh et al., 2021), and our findings extend this observation to the pre-diabetic range of insulin resistance in non-diabetic individuals.

The cerebellum is increasingly recognized for its role in cognitive functions beyond motor coordination, including executive function, working memory, and language processing (Schmahmann, 2019). The cerebellar involvement observed in our study may, therefore, have implications for understanding the cognitive consequences of metabolic dysfunction, although this requires confirmation in studies incorporating cognitive assessments.

We also observed associations between insulin resistance and reduced fusiform gyrus volume. The fusiform gyrus is involved in complex visual processing including face recognition. While this region has not been a primary focus of research on metabolic brain effects, the finding may reflect broader temporal lobe vulnerability that has been reported in studies of diabetes (Moran et al., 2013).

### 4.3 Sex differences

The association between insulin resistance and frontal sulcal enlargement was observed only in men, with formal interaction testing confirming significant effect modification by sex. Several factors may contribute to this sex difference. First, men in our sample had both a significantly higher mean HOMA-IR and a wider range of values than women, potentially resulting in greater cumulative exposure to insulin resistance-related effects as well as greater statistical power to detect an association within this subgroup. Second, estrogen has neuroprotective properties and enhances insulin sensitivity in the brain, which may attenuate the cerebral effects of peripheral insulin resistance in women (Rettberg et al., 2014). However, given the mean age of our cohort, most female participants were likely postmenopausal, when circulating estrogen levels are substantially reduced; menopausal status and hormone therapy use were not available in this dataset, limiting our ability to directly evaluate this explanation. Third, sex differences in body fat distribution and cardiometabolic risk profiles, with men having proportionally more visceral adiposity, may lead to differential metabolic effects on the brain that are not fully captured by HOMA-IR alone (Karastergiou et al., 2012).

Previous studies have reported greater age-related sulcal widening in men than in women (Jin et al., 2018; Liu et al., 2010), suggesting that males may be generally more vulnerable to sulcal changes. Our findings support the need for sex-specific approaches in research and clinical practice addressing the cerebral consequences of metabolic dysfunction.

### 4.4 Age-related patterns

The association between insulin resistance and frontal sulcal volume was strongest in younger and middle-aged participants and attenuated in those aged 70 years or older. This pattern may reflect a ceiling effect, whereby substantial age-related sulcal widening in the elderly obscures the additional contribution of insulin resistance. Alternatively, selective survival bias may operate if individuals most susceptible to the combined effects of aging and insulin resistance do not survive to older ages or develop conditions leading to study exclusion. These findings suggest that the optimal window for detecting and potentially intervening on insulin resistance-related brain changes may be in midlife. It should be noted, however, that the non-significant result in the oldest stratum (n = 171, p = 0.052) may partly reflect limited statistical power due to the smaller sample size in this group, and the positive point estimate with a wide confidence interval does not exclude a meaningful association in this age range.

### 4.5 Insulin resistance as a mediator of obesity–brain relationships

The cross-sectional design of this study precludes definitive conclusions regarding the direction of causality among BMI, insulin resistance, and sulcal morphology. While we interpreted the mediation pathway as BMI influencing insulin resistance, which in turn affects brain structure, reverse or bidirectional relationships cannot be excluded. For example, structural or functional brain changes affecting regions involved in autonomic regulation or reward processing could plausibly influence eating behavior, physical activity levels, or metabolic status, thereby contributing to the observed associations. Longitudinal studies with repeated measures of BMI, HOMA-IR, and brain structure are needed to clarify the temporal sequence of these relationships.

Mediation analysis revealed that while BMI was associated with frontal sulcal volume (total effect), this association was statistically accounted for by the indirect pathway through insulin resistance. The direct effect of BMI on sulcal volume was non-significant after adjustment for HOMA-IR, suggesting that the association between adiposity and sulcal morphology may be largely attributable to insulin resistance rather than other BMI-related pathways. These results are consistent with, though do not establish, a pathway in which higher BMI contributes to increased insulin resistance, which is in turn associated with sulcal enlargement.

These findings suggest that insulin resistance, rather than adiposity per se, may be the more proximal factor linking obesity to brain structural changes, although this interpretation should be confirmed in longitudinal studies. If confirmed, this would have clinical implications: interventions that improve insulin sensitivity might protect against metabolic brain changes even in the absence of substantial weight loss. Conversely, weight loss that does not improve insulin sensitivity may have limited benefit for brain health. Lifestyle modifications, exercise, and pharmacological agents targeting insulin sensitivity may, therefore, be valuable strategies for preventing obesity-related brain changes. The proportion mediated exceeded 100% (119%), which reflects a suppression effect in which the direct effect of BMI (c′ = −16.6 mm³) was opposite in sign to the indirect effect (a × b = +103 mm³). This pattern can arise in mediation models and does not indicate model instability, but reinforces the need for cautious interpretation of the mediation findings.

### 4.6 Clinical implications

Our findings have several clinical implications. First, brain structural changes associated with metabolic dysfunction appear to begin before the onset of diabetes, supporting the importance of early identification and intervention in individuals with insulin resistance. Second, frontal sulcal morphology may have potential as an imaging marker for detecting metabolic effects on the brain. However, the clinical and cognitive significance of the observed volumetric differences remains to be established, as the present study did not include cognitive assessment; validation in longitudinal studies incorporating cognitive outcomes is needed before frontal sulcal morphology can be considered a clinically meaningful biomarker. Third, the identification of insulin resistance as the key mediator linking obesity to brain changes suggests that interventions targeting insulin sensitivity should be prioritized. Fourth, the observed sex differences and age-related patterns indicate that middle-aged men may represent a particularly important target population for preventive strategies.

The attenuated association observed when restricting the analysis to participants with normal BMI should be interpreted with caution. This attenuation likely reflects reduced statistical power and a restricted range of HOMA-IR values within this subgroup, rather than evidence for a BMI-dependent effect of insulin resistance on sulcal morphology. The restricted range of HOMA-IR among normal-BMI participants limits the ability to detect an association even if the underlying biological effect is present across the full range of adiposity.

### 4.7 Limitations

Several limitations should be acknowledged. First, the cross-sectional design precludes causal inference. We cannot determine whether insulin resistance precedes sulcal enlargement or whether the two develop in parallel due to shared underlying factors. Longitudinal studies are needed to establish the temporal sequence and to determine whether changes in insulin resistance predict subsequent brain structural changes. Second, our sample was predominantly male (71%) and consisted entirely of adults recruited from a voluntary health checkup center, which may limit generalizability to women, other ethnic populations, and the broader general population. Individuals who elect to undergo voluntary comprehensive health examinations may differ systematically from the general population, and this should be considered when interpreting the generalizability of our findings. The sex-specific findings in particular require replication in cohorts with more balanced sex distributions. Third, residual confounding by unmeasured lifestyle factors such as diet, physical activity, and sleep quality cannot be excluded. These factors are associated with both insulin resistance and brain health, and their potential confounding effects warrant consideration. Fourth, data on menopausal status and hormone therapy use were not available, limiting our ability to evaluate the contribution of estrogen-related mechanisms to the observed sex differences. Fifth, cognitive assessment data were not available for the present cohort, precluding direct evaluation of whether the observed structural differences are associated with clinically meaningful cognitive impairment. A cognitive screening dataset existed within our institution, but only one participant overlapped with the neuroimaging cohort, making any brain–cognition association analysis statistically uninformative. Future studies incorporating concurrent cognitive assessment in the same individuals undergoing brain MRI are needed to establish the functional relevance of insulin resistance-related sulcal enlargement. Sixth, 172 participants with available MRI data were excluded due to missing HOMA-IR data, as these individuals had not undergone fasting blood sampling as part of their selected health examination package, reflecting participant choice of examination content rather than investigator-driven selection. Compared with included participants, excluded participants were older (63.7 ± 11.4 vs. 61.4 ± 11.6 years, p = 0.027), had higher BMI (28.7 ± 4.5 vs. 23.4 ± 3.5 kg/m², p < 0.001), and included a lower proportion of men (62.1% vs. 71.2%, p = 0.027). Although frontal sulcal volumes did not differ significantly between excluded and included participants (left: p = 0.793; right: p = 0.846), the excluded group nonetheless differed significantly in age, BMI, and sex distribution, and HOMA-IR—the exposure of interest—was by definition unavailable in these individuals. Selection bias related to missing HOMA-IR data, therefore, cannot be fully excluded; the absence of a difference in frontal sulcal volumes reduces, but does not eliminate, this concern. Seventh, a further limitation relates to the shared derivation of regional sulcal volumes and estimated total intracranial volume (eTIV) from the same OpenMAP-T1 segmentation. Because eTIV was calculated as the sum of all 144 regional volumes, including the sulcal regions of interest, segmentation errors could in principle propagate into both the dependent variable and this covariate. However, validation of OpenMAP-T1 against expert manual labels has shown strong volumetric agreement (Pearson and Spearman correlations >0.95) across regions, with the original validation study reporting weaker performance specifically in measures of CSF-related and sulcal structures relative to cortical and white matter regions due to greater inter-individual variability in their boundaries (Nishimaki et al., 2024). Although eTIV represents a sum across 144 regions, so that random errors in individual structures may partially cancel, this does not guard against correlated measurement error shared between the frontal sulcal volumes and eTIV, and we cannot exclude the possibility that such error influenced our results. As an independently derived intracranial volume estimate was not available in the present study, this potential source of bias could not be directly evaluated and warrants examination in future studies using alternative intracranial volume measures. Eighth, although we were able to adjust for several lifestyle-related factors in a sensitivity analysis, data on dietary intake and socioeconomic status were not available and could not be incorporated into our analyses.

## 5 Conclusions

In conclusion, this study demonstrates that sulcal morphology can detect subtle metabolic effects on brain structure at a pre-diabetic stage. Specifically, insulin resistance was associated with frontal sulcal enlargement and reduced cerebellar gray matter volume—changes that underscore the value of sulcal measures for identifying early brain vulnerability to metabolic dysfunction. The frontal sulcal association was observed only in men and was strongest in younger and middle-aged participants. Importantly, the relationship between BMI and brain structure was largely accounted for by the indirect pathway through insulin resistance, suggesting that insulin resistance rather than adiposity may be the more proximal factor. These findings suggest that metabolic effects on brain structure may be detectable in the pre-diabetic state through sulcal morphology, and that interventions targeting insulin sensitivity in midlife may help preserve brain health.

## Supplementary Material

Supplementary Material

## Data Availability

The data that support the findings of this study are not publicly available due to ethical restrictions and patient privacy considerations. Access to the data requires a formal data sharing request to the corresponding author, approval from the Institutional Review Board of The Jikei University School of Medicine, and agreement to a data use agreement. Requests may be directed to the corresponding author at d.marumaru@gmail.com.
