# Supplementary material for: Insulin resistance and brain sulcal morphology in non-diabetic adults
Source: Imaging Neurosci (Camb). 2026 Oct 16;4:IMAG.a.1402. doi: 10.1162/IMAG.a.1402 (PMC13681054; doi:10.1162/IMAG.a.1402)
Supplement: Supplementary file 1 [file IMAG.a.1402_supp.pdf]

Insulin Resistance and Brain Sulcal Morphology in Non-Diabetic Adults

Supplementary Materials

SUPPLEMENTARY FIGURE S1. ASSOCIATION BETWEEN HOMA-IR AND CEREBROSPINAL FLUID-RELATED BRAIN VOLUMES. 2

SUPPLEMENTARY FIGURE S2. AGE-STRATIFIED ASSOCIATION BETWEEN HOMA-IR AND FRONTAL SULCAL VOLUME 3

SUPPLEMENTARY TABLE S1. DISTRIBUTION OF HOMA-IR BY BMI CATEGORY 4

SUPPLEMENTARY FIGURE S3. REPRESENTATIVE EXAMPLE OF WHOLE-BRAIN PARCELLATION USING OPENMAP-T1. 4

Supplementary Figure S1. Association between HOMA-IR and cerebrospinal fluid-related brain volumes.

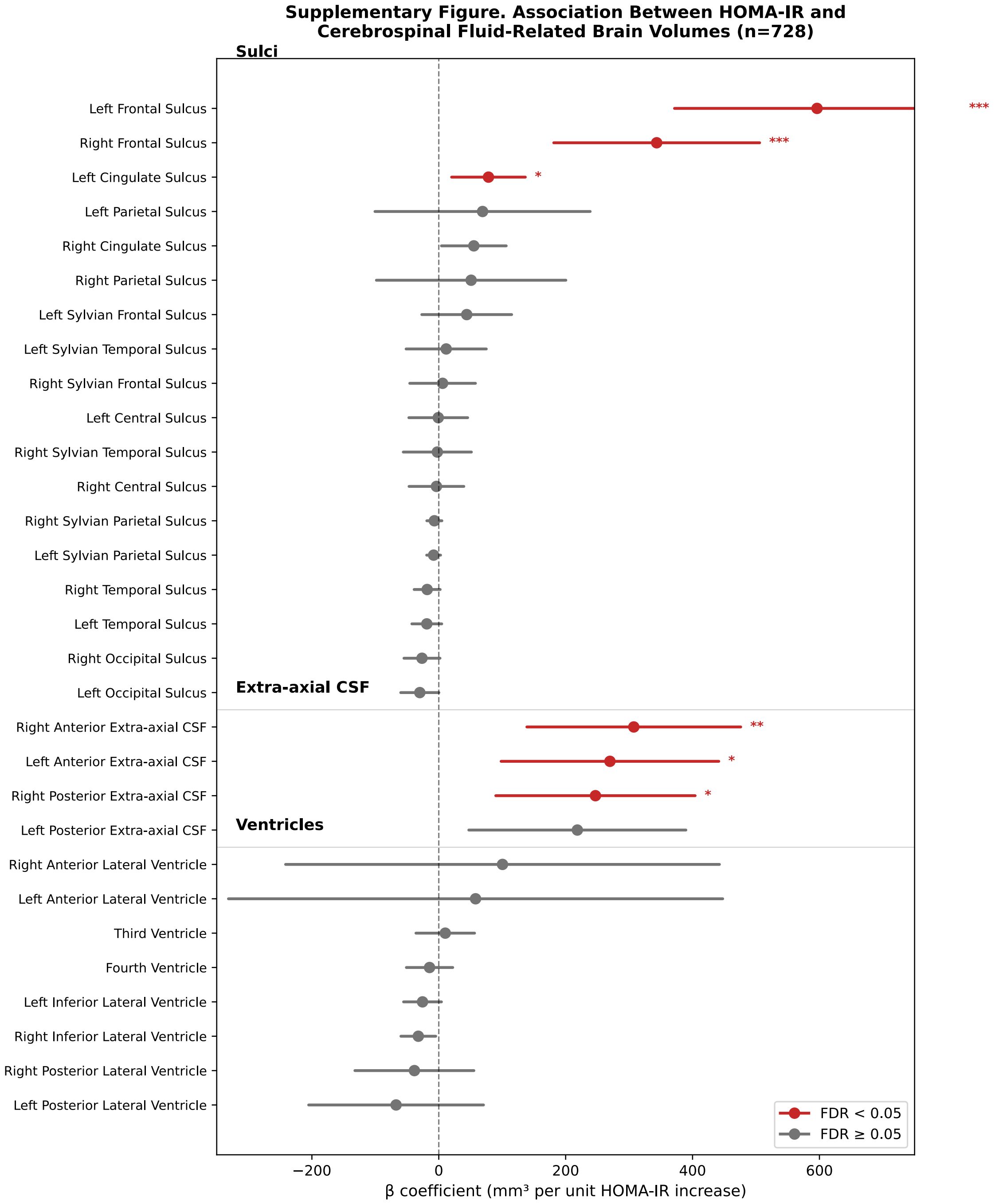

Forest plot showing β coefficients (with 95% confidence intervals) for the association between HOMA-IR and 30 CSF-related brain regions, including sulci, extra-axial CSF spaces, and ventricles.

Regions are grouped by anatomical category. Red indicates statistically significant associations after FDR correction (FDR < 0.05); gray indicates non-significant associations. Models were adjusted for age, sex, and estimated total intracranial volume. ***p < 0.001; **p < 0.01; *p < 0.05.

Supplementary Figure S2. Age-stratified association between HOMA-IR and frontal sulcal volume.

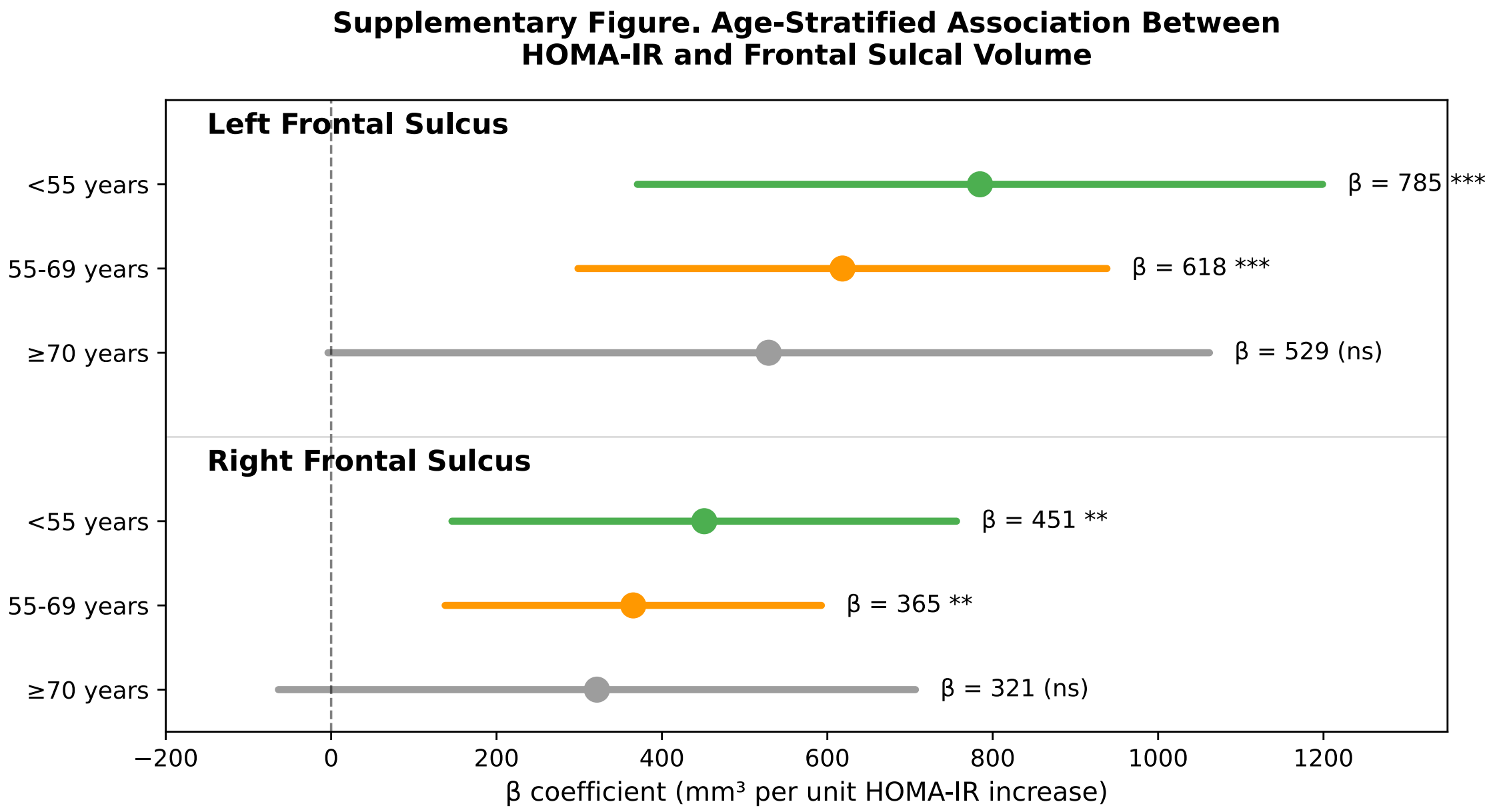

Forest plot showing β coefficients (with 95% confidence intervals) for the association between HOMA-IR and frontal sulcal volume, stratified by age group: <55 years (n = 188), 55–69 years (n = 369), and ≥70 years (n = 171). Associations were significant in younger and middle-aged participants but attenuated in older participants (≥70 years). Models were adjusted for sex and estimated total intracranial volume. ***p < 0.001; **p < 0.01; ns, not significant.

Supplementary Table S1. Distribution of HOMA-IR by BMI Category

Variable	Normal BMI (<25 kg/m², n = 511)	Overweight/Obese (≥25 kg/m², n = 217)
**HOMA-IR, mean ± SD**	1.05 ± 0.57	2.00 ± 1.15
**Insulin resistant (≥2.5), n (%)**	14 (2.7)	55 (25.3)

BMI, body mass index; HOMA-IR, homeostatic model assessment for insulin resistance; SD, standard deviation.

Supplementary Figure S3. Representative example of whole-brain parcellation using OpenMAP-T1.

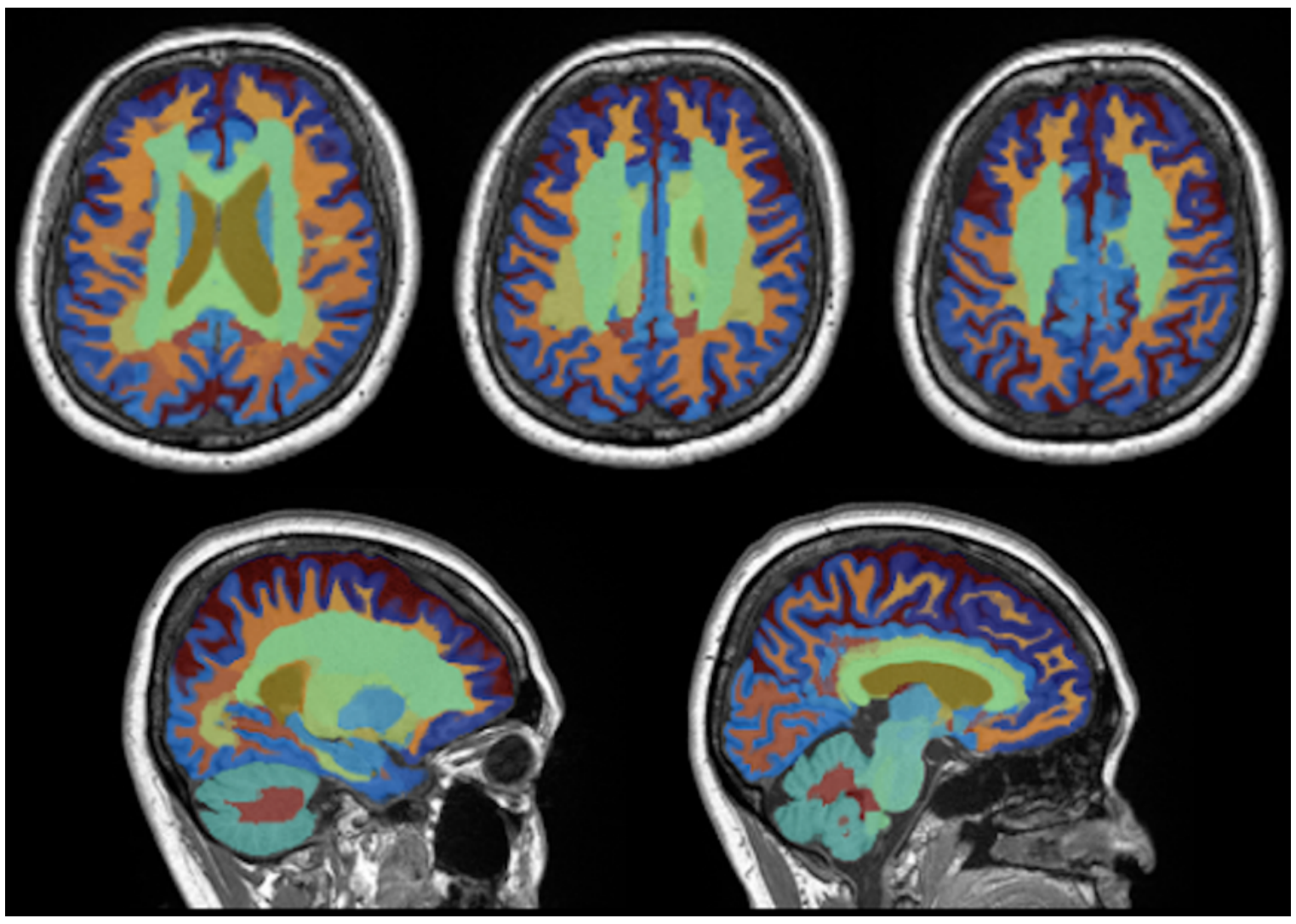

Axial (top row) and sagittal (bottom row) views of T1-weighted MRI overlaid with OpenMAP-T1 parcellation output from a representative participant, illustrating anatomically plausible segmentation of cortical, subcortical, ventricular, and cerebrospinal fluid-related structures, including the frontal sulci. The frontal sulci, the primary imaging outcome of this study, are visible bilaterally in both the axial and sagittal views, allowing visual confirmation of their segmentation.
